# Child and Parent Physical Activity, Sleep, and Screen Time During COVID-19 and Associations With Mental Health: Implications for Future Psycho-Cardiological Disease?

**DOI:** 10.3389/fpsyt.2021.774858

**Published:** 2022-02-10

**Authors:** Lisa S. Olive, Emma Sciberras, Tomer S. Berkowitz, Erin Hoare, Rohan M. Telford, Adrienne O'Neil, Antonina Mikocka-Walus, Subhadra Evans, Delyse Hutchinson, Jane A. McGillivray, Michael Berk, Sam J. Teague, Amanda G. Wood, Craig Olsson, Elizabeth M. Westrupp

**Affiliations:** ^1^Centre for Social and Early Emotional Development, School of Psychology, Deakin University, Canberra, VIC, Australia; ^2^Faculty of Health, The Institute for Mental and Physical Health and Clinical Translation (IMPACT), Deakin University, Geelong, VIC, Australia; ^3^Orygen, Elite Sports and Mental Health, and the Department of Psychiatry, The University of Melbourne, Parkville, VIC, Australia; ^4^Murdoch Children's Research Institute, Parkville, VIC, Australia; ^5^Research Institute for Sport and Exercise, University of Canberra, Canberra, VIC, Australia; ^6^The Food and Mood Centre, Deakin University, Geelong, VIC, Australia; ^7^The National Drug and Alcohol Research Centre, University of New South Wales, Sydney, NSW, Australia; ^8^Florey Institute of Neuroscience and Mental Health, The University of Melbourne, Melbourne, VIC, Australia; ^9^College of Health & Life Sciences, Aston University, Birmingham, United Kingdom; ^10^Department of Paediatrics, University of Melbourne, Melbourne, VIC, Australia; ^11^Judith Lumley Centre, La Trobe University, Melbourne, VIC, Australia

**Keywords:** psychiatry, physical activity, children, adults, depression, anxiety, sleep, screen time

## Abstract

The COVID-19 pandemic has afforded the opportunity for some to improve lifestyle behaviours, while for others it has presented key challenges. Adverse changes in global lifestyle behaviours, including physical activity, sleep, and screen time can affect proximal mental health and in turn distal cardiovascular outcomes. We investigated differences in physical activity, sleep, and screen time in parents and children during early stages of the COVID-19 pandemic in Australia compared to pre-COVID-19 national data; and estimated associations between these movement behaviours with parent and child mental health. Cross-sectional baseline data from the COVID-19 Pandemic Adjustment Study (CPAS; *N* = 2,365) were compared to nationally representative pre-pandemic data from the Longitudinal Study of Australian Children (LSAC; *N* = 9,438). Participants were parents of children aged ≤ 18 years, residing in Australia. Parents provided self-report measures of mental health, physical activity and sleep quality, and reported on child mental health, physical activity and screen time. Children in CPAS had significantly more sleep problems and more weekend screen time. Their parents had significantly poorer sleep quality, despite increased weekly physical activity. Children's sleep problems were significantly associated with increased mental health problems, after accounting for socioeconomic status, physical activity, and screen time. Poorer parent sleep quality and lower levels of physical activity were significantly associated with poorer mental health. Monitoring this cohort over time will be important to examine whether changes in movement behaviour are enduring or naturally improve with the easing of restrictions; and whether these changes have lasting effects on either parent or child mental health, and in turn, future risk for CVD.

## Introduction

COVID-19, a disease caused by the Severe Acute Respiratory Syndrome Coronavirus 2 (SARS-Cov-2), was classified as a pandemic by the World Health Organization on March 11, 2020 (1). In an attempt to stop the spread of COVID-19, strict quarantine, lockdown, and social distancing measures were implemented around the world, including in Australia, where Federal and State governments introduced Stage three lockdown (2) in April 2020 to reduce the rate of infection. Measures to prevent the spread of COVID-19, such as home confinement, school closures, restriction on the gathering of groups of people, and the shutdown of community services and facilities (e.g., organised sports, playgrounds, pools, and gyms) are likely to have an impact on a number of important health promoting global movement behaviours [e.g., physical activity, screen time and sleep; (3, 4)]. These behaviours are closely related to both mental health and risk for cardiovascular disease (CVD). Given that the childhood origins of mental health disorders and CVD are increasingly recognised (5), and there are known links between depression, stress and risk for CVD (6–8), there is potential that adversity associated with the COVID-19 pandemic may have long-term effects on the development of psycho-cardiologic diseases. This is plausible due to the shared risk pathways that have been shown to link a range of mental health indicators (e.g., depression, psychosocial stress, lack of social support and negative emotions) with CVD, including both intermediary markers of CVD risk (9, 10) and established CVD [(11); for a review of shared pathways see (12)].

Emerging evidence on how COVID-19 related restrictions may be affecting people's physical activity, sleep quality and screen time has been mixed (13–16) but it is not difficult to see how such restrictions may have a negative impact across these domains. For example, the closure of parks, beaches and sports facilities, a lesser need for active transport as people are urged to stay home and increases in screen use related to working and schooling from home, are likely to reduce the time that children and parents spend in environments associated with physical activity. This may be offset at least in part by physical activity being one of a very small number of permitted activities. Similarly, increased time at home may disrupt normal routines, with increased access to screens and potential changes to bedtime routines. In addition, pre-pandemic research has shown that, on unstructured and non-school days (i.e., similar to COVID-19-related home confinement), children are less physically active and more sedentary (17), engage in more recreational screen time (18) and have less regularity in sleep patterns (19). To-date, there has been minimal evidence considering the impact of home confinement on physical activity, sleep and screen time for both children and parents within households, meaning that the overall family burden of changes in movement related behaviours is not well-understood. A family systems perspective on the impact of COVID-19 on movement behaviours may be a novel and conceptual approach, particularly in relation to understanding mental health within families.

Decreased physical activity and increased sedentary behaviour may be a risk factor not only for more proximal indicators of mental health but in turn, for more distal risk of CVD among both children and their parents (20, 21). For example, poor sleep is closely linked with symptoms of depression and anxiety in children (22) as well as in adults (23, 24). Similarly, lower levels of physical activity and greater amounts of time spent in sedentary activities are linked to poorer mental health outcomes among children, including anxiety and depressive symptoms (25, 26) and irritability (27). A similar picture presents itself in adults, where reductions in physical activity are related to increases in mental health symptoms (28, 29) and a greater likelihood of a clinical diagnosis, such as major depressive disorder (30, 31). Furthermore, any negative change to movement behaviours, brought about by COVID-19 restrictions, may be further compounded by COVID-19 stressors, including educational (school disruption), environmental (e.g., loss of employment, housing) and biopsychosocial (e.g., COVID-19 diagnosis, loss of social support) stressors. Within families, these have the potential to directly impact child health through adverse impacts on parent health. Given that poor mental health is also a known risk factor for CVD, alongside these lifestyle risk factors, gaining a clear understanding of the mental health burden of the COVID-19 pandemic among families and its relationship to global movement behaviours can inform targeted public health efforts for effective prevention and/or intervention.

The aim of the current study was to investigate levels of physical activity, screen time, and sleep during the early stages of COVID-19 social restrictions in Australia, and compare it to pre-COVID-19 national data. Further, we sought to estimate the association between these movement behaviours and a range of mental health indicators among both parents and children. Specific aims were to:

- Compare levels of child screen time, sleep problems and regular bedtime routine during the early stages of the COVID-19 social restrictions to Australian pre-pandemic national data.
- Compare levels of parent sleep quality and physical activity during the early stages of the COVID-19 pandemic to Australian pre-pandemic national data.
- Investigate the associations between child physical activity, sleep and screen time with child indicators of mental health, including depression symptoms (in children aged 2–18 years), anxiety symptoms (in children aged 2–18 years) and irritability (in children aged 0–18 years), and whether these associations were moderated by COVID-19 related stressors.
- Investigate the association between parent physical activity and sleep quality with parent depression, anxiety, stress, emotion regulation and positive affect, and whether these associations were moderated by COVID related stressors.

## Methods

### The COVID-19 Pandemic Adjustment Survey (CPAS)

This study uses cross-sectional baseline data from the COVID-19 Pandemic Adjustment Survey (CPAS) (32), which aims to investigate the mental health impacts of the COVID-19 crisis in a sample of Australian parents using regular (longitudinal) online surveys. Participants were recruited via paid and unpaid social media advertisements using a range of methods to increase the representativeness of the sample (e.g., targeting demographic factors/postcodes). Recruitment occurred from 8 to 28th April, 2020, which coincided with the Australian federal and state governments introducing increasingly strict social distancing measures to slow the rate of infection. Participants were 2,365 Australian parents. Parents were eligible to participate if they resided in Australia and were aged ≥18 years, English speaking and a current parent of a child aged 0–18 years. The current study was approved by the Deakin University Human Ethics Advisory Group (HEAG-H 52_2020).

### The Longitudinal Study of Australian Children (LSAC)

As a means of pre-pandemic comparison, we drew on national data from the Longitudinal Study of Australian Children (LSAC), a nationally-representative prospective cohort study of Australian children and their families (33). Participating children were randomly selected in a two-stage cluster sampling design using Australian postcodes and the Medicare universal healthcare database (34). We used biennial parent-report data (99% mothers) from three waves of the Baby cohort (*N* = 5,107), covering child ages 0–1 (Wave 1, collected in 2004), 2–3, and 4–5 years; and from five waves of the Kindergarten cohort (4,656), covering child ages 6–7 (Wave 2, collected in 2006), 8–9, 10–11, 12–13, and 14–15 years.

### Measures

Table 1 provides a summary of the socio-demographic, parent, child and COVID-19 risk measures from CPAS and LSAC used in the current analysis.

**Table 1:** Study measures.

**Construct**	**Measure (items)**	**CPAS**	**LSAC**
Demographics	Parent age, gender, country of birth, Aboriginal and Torres Strait Islander status, whether a language other than English was spoken at home, highest level of education, relationship status, whether they were living with their partner, number of children in the household, geographic location and whether they had a mental health or physical health condition. Demographic information prior to COVID-19 included employment and study status, household income, source of income and shortage of money. Parents also reported on their child's age, gender and whether their child had a neurodevelopmental condition (attention-deficit/hyperactivity disorder, autism spectrum disorder).	•	
COVID-19 psychological stressors	4 items adapted from the CoRonavIruS Health Impact Survey (CRISIS) V0.1. (35). Two items asked about participants' feelings about the likelihood catching COVID-19, and COVID-19 being a serious health risk for the participant (both rated on a 7-point Likert scale ranging from 1 = Strongly Disagree to 7 = Strongly Agree) and two items asked about the extent to which they experience certain negative feelings (worry, fear) when thinking about their ability to deal with COVID-19 (both items rated on a 4-point scale from “not at all” to “a great deal”). These items were averaged to from a total score (range, 3–18), with higher scores reflecting greater COVID-19 related psychological stress (α = 0.69).	•	
COVID-19 environmental stressors	The following binary coded stressors (presence/absence of stressor) were summed to form a count variable (range, 0–6). Housing insecurity, financial insecurity; job loss, new job, reduction or increases in work hours, or changes (“redeployment”) in employment; food shortages; and COVID-19 illness (contracting the COVID-19 virus, hospitalisation of themselves or a family member due to infection, and death of a family member due to the virus).	•	
**Parent measures**		
Mental health	Depression Anxiety Stress Scale (DASS) - Symptoms of depression, anxiety and stress in the last week were assessed using the widely used and well-validated Depression Anxiety Stress Scale-21 [DASS-21; (36–38)]. The scale comprises three 7-item subscales to measure depression (α = 0.89), anxiety (α = 0.82), and stress (α = 0.87), with items rated on a 4-point Likert scale (0 = Did not apply to me at all to 3 = Applied to me very much, or most of the time). Higher scores indicate greater symptom severity.	•	
Emotion regulation	Difficulties in Emotion Regulation Scale-16 Item Version - Used to assess emotion regulation in parents (α = 0.95). Each item is rated on a 5-point Likert scale ranging from 1 = Almost never to 5 = Almost always (no time frame specified). Higher scores indicate greater emotion regulation difficulties.	•	
Positive affect	Positive and Negative Affect Schedule Short Form - A valid and reliable (39) 5-item measure whereby participants rated on a 5-point Likert scale (1 = Very Slightly or Not at All to 5 = Extremely) whether each affective state applies to them in the present moment (α = 0.80). Higher scores indicate greater positive affect.	•	
Physical activity	Parent physical activity was assessed using the LSAC physical activity item, “About how many days each week do you do at least 30 min of moderate or vigorous physical activity (like walking briskly, riding a bike, gardening, tennis, swimming, running, etc.?)”, rated from 1 to 7 days.	•	•
Sleep	Parent sleep quality was assessed using the LSAC item, “During the past month, how would you rate your sleep quality overall?” Rated on a 4-point scale from “very good” to “very bad”. Item responses were then dichotomised, where response options 1–2 were coded 1 (very good/good) and 3–4 were coded 2 (very bad/bad).	•	•
**Child measures**		
Depression^a^	Short Mood and Feelings Questionnaire (SMFQ) – 13-item measure of child depression symptoms with items rated on a 3-point Likert scale (1 = Not true to 3 = True) (overall α = 0.87; children 6 years and above, α = 0.88; children <6 years, α = 0.79). Higher scores indicate greater depression symptoms.	•	
Anxiety^a^	Modified Brief Spence Children's Anxiety Scale – 4-items assessing symptoms of anxiety (α = 0.77). Parents rated on a 4-point Likert scale (“never” to “always”) how frequently their child had experienced symptoms in the last 2 weeks. Higher scores indicate greater anxiety symptoms.	•	
Irritability	1 item, adapted from the CoRonavIruS Health Impact Survey (CRISIS) (35), which asked, “During the past 2 weeks, how irritable or easily angered has your child been?” Parents rated this item on a 5-point Likert scale, ranging from “Not at all” to “Extremely,” with higher scores indicating greater child irritability.	•	
Physical activity	Assessed via 1 item, adapted from LSAC, which asked parents, “About how many days each week did your child do at least 30 min of moderate or vigorous physical activity (like walking briskly, riding a bike, gardening, tennis, swimming, running, etc.?)” Parents provided a rating ranging from 0 to 7 days.	•	•
Sleep	Sleep pattern was assessed using the LSAC item, “How much is your child's sleeping pattern or habits a problem for you?” rated on a 4-point scale (“not at all a problem” to “a large problem”). Responses dichotomised, where response options 1–2 were coded 1 (not a problem/small problem) and response options 3–4 were coded 2 (moderate problem/large problem) as per previous research (40, 41). Child sleep regularity was assessed using the LSAC item, “Does the study child go to bed at regular times?” Parents rated this item on a 5-point scale, ranging from “never” to “always.” Item responses were then dichotomised, where response options 1–3 (never/rarely/sometimes) were coded 1 and response options 4–5 (usually/always) were coded 2, with higher scores representing a more regular bedtime.	•	•
Screen time	Two items from LSAC, which asked how many hours on a typical (item 1) weekday and (item 2) weekend day did their child use media devices at home for non-educational purposes. Each item was rated on a sliding scale from 1 to 24 hours and provided a measure of weekday screen time and weekend day screen time (hours per day).	•	•

*CPAS, COVID-19 Pandemic Adjustment Survey; LSAC, Longitudinal Study of Australian Children.*

a *Measured only in children aged ≥2 years of age*.

#### Population Weighting

Post-stratification weights in the CPAS dataset were derived to compensate for differences between the final sample and the national population of parents. Post-stratification weights for CPAS were produced through a raking approach (42), using six demographic factors: (1) geographic location (major city, inner and outer regional areas, and remote areas); (2) child age groups (0–4, 5–9, 10–12, 13–14, and 15–18 years); (3) parent gender (male, female); (4) family structure (single parent, couple family); (5) parent education (did not complete high school, high school completion; and (6) pre-pandemic parent employment status (employed, unemployed). The CPAS dataset was weighted to be equivalent to a subpopulation of Australian adults (i.e., parents of a child 0–18 years), with an estimated total population size of 8.4 million parents. LSAC population weights were also applied in weighted analyses.

### Statistical Analysis

Analyses were conducted in Stata version 16. Item level missing data in the CPAS study ranged from 0 to 8% on individual variables; and up to 29% in the LSAC study. Missing data were therefore replaced using multivariate multiple imputation by chained equations for each study separately. All variables from the final analytic models and weights were included in the multiple imputation model to create 50 imputed datasets. All reported results are from the multiply imputed datasets.

To address Aims 1 and 2, proportions and 95% confidence intervals were calculated for LSAC data using all available time-points for each measure. Two sets of CPAS estimates were calculated; firstly, for the full available sample; and secondly, for an age-matched subsample consistent with LSAC age ranges to allow comparison with the LSAC national data. LSAC estimates were averaged across available waves within each imputation model, and then combined across the Baby and Kindergarten cohorts using a meta-analysis process based on metaphor in R (43, 44). For CPAS and LSAC, both unweighted and weighted data are presented to ensure reported outcomes are as close to population representation as possible. However, given that our datasets varied from population statistics on several population indicators (see Table 2), we primarily focused on unweighted results. We conducted a series of independent samples *t*-tests to assess unweighted differences between samples/groups.

**Table 2:** Participant characteristics in the COVID-19 Pandemic Adjustment Survey (*N* = 2365, child aged 0–18 years) and the Longitudinal Study of Australian Children Baby Cohort (Wave 1, *N* = 5,107) and Kindergarten Cohort (Wave 3, *N* = 4,331).

**Participant characteristic^a^**	**CPAS**	**LSAC-B**	**LSAC-K**
	**(Time 1, 2020)**	**(Wave 1, 2004)**	**(Wave 3, 2008)**
Parent age, m(sd)	38.30 (7.07)	31.0 (5.51)	39.1 (5.40)
Child age, m(sd)	8.66 (5.14)		8.26 (0.44)
**Parent gender**			
Cisgender men	19.2%	1.5%	4.3%
Cisgender women	80.7%	98.6%	95.8%
Transgender or non-binary	<1.00%	NA	NA
**Child gender**			
Cisgender boy	50.9%	51.1%	51.1%
Cisgender girl	48.7%	48.9%	49.0%
Transgender or non-binary	<1.0%	NA	NA
Aboriginal or Torres Strait Islander	2.1%	NA	NA
Language other than English	4.5%	14.4%	13.7%
Parent born overseas	17.9%	18.6%	20.4%
Low household income^b^	14.0%	NA	NA
Receiving government benefit	5.8%	NA	NA
Did not complete high school	9.4%	33.3%	39.1%
Single parent household	10.9%	9.5%	29.3%
**Geographic location**			
Major cities of Australia	69.8%	NA	NA
Inner regional Australia	23.2%	NA	NA
Outer regional Australia	6.1%	NA	NA
Remote Australia	1.0%	4.3%	3.7%
**Number of children**			
1 child	28.4%	39.5%	8.2%
2 children	46.1%	36.7%	44.2%
3 children	18.2%	16.1%	31.3%
4 or more children	5.3%	7.6%	16.4%
Parent mental or physical health condition	56.1%	NA	NA
**Child neurodevelopmental condition**			
Attention-deficit/hyperactivity disorder	7.3%	NA	NA
Autism spectrum disorder	8.6%	NA	NA
**COVID-19 related factors**			
Deprivation index, m(sd)	0.38 (0.95)	NA	NA
Child home while working	49.64%	NA	NA
Parent reported CVD	0.9%	NA	NA

*m(sd), Mean (standard deviation), data are unweighted; NA, not applicable.*

a *Data collected 8th−28th April 2020, data are multiply imputed and thus n cannot be presented for each characteristic.*

b *$52,000 or less per year*.

To address Aims 3 and 4, a series of unadjusted linear regression models, using unweighted CPAS data, were conducted to determine the association between movement behaviours and indicators of mental health in the CPAS sample. Next, adjusted models were conducted to assess the unique contribution of each movement behaviour to each indicator of mental health, while accounting for the contribution of the other concomitant variables in the model. In regression models assessing adult data, adjustments were made for age, gender, deprivation, education, Aboriginal and Torres Strait Islander Peoples status and language other than English (LOTE) spoken at home, and for data related to child factors, adjustments were made for child age, child gender, parent age, parent gender, partner status, deprivation, and parent education. Final adjusted models were derived by including concomitant variables where there was evidence for an unadjusted association with the outcome (*p* < 0.1). Interaction terms were added to assess whether COVID related stressors moderated relationships between the movement behaviours and mental health indicators.

## Results

### Participant Characteristics

Participant characteristics are presented in Table 2. CPAS parents had a mean age of 38 years (*SD* = 7.07), were primarily female (81%), resided in a major city (70%) and had two children (46%) who were on average 9 years old (*SD* = 5.14). Single parent households were a minority (11%), and half of the sample had a child at home while working (50%). Twelve percent of parents reported that their child had an attention-deficit/hyperactivity disorder (ADHD) and/or autism spectrum disorder (ASD) and more than half of parents indicated that they themselves had either a physical or mental health condition. In contrast, LSAC parents in both cohorts were almost all female, but there were similar proportions of LSAC parents born overseas or from single parent families, compared to CPAS parents. A greater proportion of LSAC parents spoke a language other than English and did not complete high school.

### Physical Activity, Screen Time, and Sleep During COVID-19 Compared to National Data

Table 3 presents parent and child physical activity, sleep, and screen time for CPAS compared to LSAC data.

**Table 3:** Parent and child physical activity, sleep, and screen time compared to Australian normative data (LSAC).

	**Unweighted**						**Weighted**					
	**CPAS**			**LSAC**			**CPAS**			**LSAC**		
	**%**	**LL**	**UL**	**%**	**LL**	**UL**	**%**	**LL**	**UL**	**%**	**LL**	**UL**
**Child sleep problem**												
Full CPAS sample	17.7	15.9	19.4				16.4	13.6	19.2			
Matched: children 0–13 years	17.4	15.5	19.3	8.9	8.6	9.3	16.9	13.6	20.2	9.6	9.1	10.0
**Child no regular bedtime**												
Full CPAS sample	20.1	18.3	21.8				21.5	18.2	24.9			
Matched: Children 0–13 years	13.1	11.4	14.8	2.8	2.5	3.0	15.0	11.3	18.7	3.1	2.7	3.4
**Parent poor sleep quality**												
Full sample	56.0	53.9	58.1				58.3	54.4	62.2			
Matched: Children 0–13 years	56.7	54.3	59.0	21.0	20.4	21.6	59.1	54.6	63.5	21.6	21.0	22.3
	**M (se)**	**LL**	**UL**	**M (se)**	**LL**	**UL**	**M (se)**	**LL**	**UL**	**M (se)**	**LL**	**UL**
**Screen-time (weekday)**												
Full CPAS sample	3.19 (0.07)	3.05	3.33				3.56 (0.13)	3.31	3.82			
Matched: Children 4–13 years	2.84 (0.07)	2.70	2.99	2.98 (0.01)	2.97	2.99	3.14 (0.13)	2.89	3.39	3.02 (0.01)	3.01	3.04
Screen-time (weekend)												
Full CPAS sample	4.33 (0.08)	4.18	4.48				4.84 (0.14)	4.56	5.11			
Matched: Children 4–13 years	3.98 (0.08)	3.82	4.13	3.35 (0.01)	3.34	3.37	4.45 (0.17)	4.12	4.79	3.36 (0.01)	3.34	3.38
Child physical activity												
CPAS: Children 1–18 years	4.93 (0.05)	4.84	5.02				4.91 (0.08)	4.75	5.07			
**Parent physical activity**												
Full CPAS sample	3.88 (0.04)	3.80	3.97				4.03 (0.09)	3.85	4.22			
Matched: Children 0–13 years	3.86 (0.05)	3.77	3.96	2.85 (0.01)	2.82	2.88	4.04 (0.11)	3.82	4.25	2.85 (0.02)	2.81	2.88

*“Full CPAS sample” refers to children aged 0–18 years. “Matched” samples refer to CPAS participants that match the child age range available for LSAC normed data. LL/UL refer to the lower limit (LL) and upper limit (UL) estimates from 95% confidence intervals. se, standard error*.

#### Child

During the pandemic, ~1 in 6 parents reported that their child's sleep was a problem, in both the full CPAS sample (i.e., children 0–18 years) and the matched CPAS sample (children 0–13 years). This rate was close to double the rates reported by LSAC parents of children 0–13 years [17.4 vs. 8.9%, $X_{\left(1,11,621\right)}^{2}$ = 109.23, *p* < 0.001]. Although 1 in 5 parents reported their child had no regular bedtime in the full CPAS sample, this was less frequent in the age-matched sample, where approximately 1 in 8 children did not have a regular bedtime. However, this rate was still much higher than the LSAC national data [13.1 vs. 2.8%, $X_{\left(1,\;11,621\right)}^{2}$ = 79.49, *p* < 0.001].

In the CPAS full sample, children spent on average just over 3 and 4 h per day, respectively, engaged in weekday, and weekend day recreational screen time. These times were lower in the CPAS age-matched sample (i.e., aged 4–13 years), and there was evidence that CPAS parents reported their child spent less time engaged in recreational screen time on weekdays [*t*_(11, 183)_ = 4.31, *p* < 0.001, *d* = 0.12] but more time on weekend days [*t*_(11619)_ = −16.72, *p* < 0.001, *d* = −0.42] compared to the LSAC national data.

#### Parents

On average, parents participated in at least 30 min of physical activity up to 4 days per week, in both the full and matched CPAS samples, which was higher than the average rates reported in the LSAC national data [*t*_(11620)_ = −24.96, *p* < 0.001, *d* = −0.63]. Over half of parents reported poor sleep quality in both the full and matched CPAS samples, which was more than double the average rates reported in LSAC national data [56.7 vs. 21%, $X_{\left(1,11,621\right)}^{2}$ = 1147.12].

#### Sensitivity Analysis

Given the high proportion of CPAS parents who reported having a child with ADHD and/or ASD, and the known links between the movement behaviours and mental health outcomes among these children and parents (45–47), a sensitivity analysis was run removing parents who reported having a child with these conditions to determine the effect on our findings (*n* = 15%). No differences were found in our findings comparing CPAS age-matched sample with and without ADHD/ASD children compared to LSAC national data, indicating this over-representation did not change the interpretation of the findings reported.

### Association Between Movement Behaviours and Mental Health Outcomes

#### Child

Unadjusted and adjusted models investigating the association between movement behaviours and mental health outcomes for children in the CPAS cohort are presented in Table 4. In unadjusted models, participating in at least 30 min of moderate or vigorous physical activity per day on fewer days (*B* = −0.22, 95% *CI* [−0.32 to −0.12], *p* < 0.001), spending more hours per day engaged in weekday (*B* = 0.26, 95% *CI* [0.20–0.32], *p* < 0.001) and weekend day (*B* = 0.25, 95% *CI* [0.19–0.31], *p* < 0.001) recreational screen time, having no regular bedtime (*B* = 1.40, 95% *CI* [0.90–1.90], *p* < 0.001) and greater sleep problems (*B* = 2.68, 95% *CI* [2.18–3.18], *p* < 0.001) were associated with greater depressive symptoms. These associations remained significant in adjusted models for weekend screen time and sleep problems (both *p* < 0.001).

**Table 4:** Associations between child movement behaviours (physical activity, screen time, sleep problems, and sleep routine) and mental health outcomes.

	**Unadjusted**				**Adjusted**			
	***Coefficient***	**LL**	**UL**	***p***	***Coefficient***	**LL**	**UL**	***p***
**Child depression^a^**								
Physical activity	−0.22	−0.32	−0.12	0.000	−0.05	−0.15	0.06	0.362
Screen time (weekday)	0.26	0.20	0.32	0.000	0.08	−0.02	0.18	0.123
Screen time (weekend)	0.25	0.19	0.31	0.000	0.13	0.03	0.23	0.009
Sleep problems	2.68	2.18	3.18	0.000	2.35	1.83	2.88	0.000
No regular bedtime	1.40	0.90	1.90	0.000	−0.08	−0.64	0.48	0.776
**Child anxiety^b^**								
Physical activity	0.03	−0.03	0.09	0.298	0.05	−0.01	0.11	0.090
Screen time (weekday)	0.02	−0.02	0.05	0.382	−0.02	−0.07	0.03	0.455
Screen time (weekend)	0.02	−0.02	0.05	0.312	0.05	−0.01	0.10	0.078
Sleep problems	1.40	1.11	1.68	0.000	1.36	1.06	1.65	0.000
No regular bedtime	0.08	−0.18	0.34	0.562	−0.30	−0.59	−0.01	0.042
**Child Irritability^c^**								
Physical activity	0.03	0.01	0.05	0.010	0.04	0.02	0.06	0.001
Screen time (weekday)	0.01	−0.01	0.02	0.229	0.01	−0.02	0.03	0.654
Screen time (weekend)	0.01	−0.01	0.02	0.375	0.02	0.00	0.05	0.057
Sleep problems	0.34	0.22	0.46	0.000	0.33	0.20	0.45	0.000
No regular bedtime	0.01	−0.10	0.13	0.809	−0.01	−0.14	0.12	0.862

a *Adjusted for child age, parent gender, no partner and other movement behaviours.*

b *Adjusted for child age, parent gender, deprivation, no partner, education and other movement behaviours.*

c *Adjusted for child age, parent age, parent gender and other movement behaviours*.

In unadjusted models, more disrupted sleeping patterns in the CPAS cohort were associated with greater child anxiety symptoms (*B* = 1.40, 95% *CI* [1.11–1.68], *p* < 0.001) but no associations were found for having no bedtime routine, weekday or weekend screen time or physical activity (all *p* > 0.05). In adjusted models, the association remained for sleep problems (*B* = 1.30, 95% *CI* [1.01–1.60], *p* < 0.001) and having no regular bedtime became significantly associated with anxiety symptoms (*B* = −0.30, 95% *CI* [−0.59 to −0.01], *p* = 0.042). Higher levels of physical activity (*B* = 0.04, 95% *CI* [0.02–0.06], *p* = 0.001) and greater sleep problems (*B* = 0.33, 95% *CI* [0.20–0.45], *p* < 0.001) were both associated with child irritability in adjusted models. There was no significant moderating effect of COVID-19 environmental stressors or COVID-19 psychological stressors on the associations between movement behaviours and child mental health outcomes (all *p* > 0.05).

##### Sensitivity Analysis

Given some measures have not yet been validated in a younger sample (e.g., our measure of depression and anxiety have been validated for children as young as 6 years, yet we investigated associations in children as young as 2 years in our full CPAS sample), we undertook a sensitivity analysis, including only children aged 6 years and above. While for the most part our finding remained unchanged, when limiting the sample to 6 years and above, having an irregular bedtime was not associated with anxiety (*p* = 0.181) and weekend screen time was not associated with depression (*p* = 0.111).

#### Parent

Unadjusted and adjusted models examining the association between movement behaviours and mental health outcomes for CPAS parents during the pandemic are summarised in Table 5. In adjusted models, lower levels of physical activity and poorer sleep quality were associated with greater parent depressive symptoms. Lower levels of physical activity and poorer sleep quality were also associated with parent anxiety and stress symptoms. Similarly, less physical activity and poorer sleep quality were associated with greater difficulties regulating emotions. More physical activity and greater sleep quality was associated with greater positive affect. COVID-19 environmental stressors moderated the association between sleep quality and depression (*B* = 0.31, 95% *CI* [0.02–0.60], *p* = 0.034), and moderated the association between sleep quality and difficulties with emotion regulation (*B* = 0.96, 95% *CI* [0.09–1.84], *p* = 0.031), where parents reporting both high COVID-19 stressors and poor sleep quality reported the highest levels of emotion regulation difficulties and depression (see Tables 1, 2). There was no significant moderating effect of COVID-19 psychological stressors on the associations between movement behaviours and parent mental health (all *p* > 0.05).

**Table 5:** Associations between parent movement behaviours (physical activity and sleep quality) and mental health outcomes.

	**Unadjusted**				**Adjusted**			
	***Coefficient***	**LL**	**UL**	***p***	***Coefficient***	**LL**	**UL**	***p***
**Depressive symptoms^a^**								
Physical activity	−0.40	−0.48	−0.31	0.000	−0.28	−0.36	−0.20	0.000
Poor sleep quality	2.63	2.29	2.98	0.000	2.16	1.82	2.51	0.000
**Anxiety symptoms^b^**								
Physical activity	−0.23	−0.30	−0.17	0.000	−0.13	−0.19	−0.07	0.000
Poor sleep quality	1.97	1.69	2.25	0.000	1.59	1.31	1.87	0.000
**Stress^c^**								
Physical activity	−0.29	−0.38	−0.21	0.000	−0.14	−0.22	−0.06	0.001
Poor sleep quality	3.56	3.21	3.91	0.000	3.18	2.83	3.53	0.000
**Emotion regulation^d^**								
Physical activity	−0.89	−1.13	−0.65	0.000	−0.56	−0.80	−0.33	0.000
Poor sleep quality	6.60	5.57	7.62	0.000	5.44	4.42	6.46	0.000
**Positive affect^e^**								
Physical activity	0.57	0.50	0.64	0.000	0.49	0.42	0.56	0.000
Poor sleep quality	−1.98	−2.29	−1.68	0.000	−1.59	−1.88	−1.29	0.000

a *Adjusted for parent age, deprivation, parent education, and other movement behaviours.*

b *Adjusted for parent age, parent gender, deprivation, parent education, parent ATSI status, and other movement behaviours.*

c *Adjusted for parent age, parent gender, deprivation, language other than English spoken at home and other movement behaviours.*

d *Adjusted for parent age, deprivation, parent education, and other movement behaviours.*

e *Adjusted for parent age, parent gender, parent Aboriginal, and Torres Strait Islander Peoples status and other movement behaviours*.

## Discussion

This study investigated differences in a set of movement behaviours, namely physical activity, sleep and screen time, in parents and children during early stages of the COVID-19 pandemic in Australia compared to pre-COVID-19 national data; and estimated associations between these movement behaviours with parent and child mental health. Given the growing body of research establishing the mental health impacts of COVID-19 (48, 49), and the known association between movement behaviours with proximal indicators of mental health and more distal risk for CVD, identifying modifiable factors that can be targeted to improve both mental health and risk for CVD, is a priority. Here we show that children and parents under lockdown restrictions report substantially higher rates of sleep problems compared to pre-pandemic rates, with parents also reporting their child had greater levels of recreation screen time on weekends. Children who participated in lower levels of physical activity, had higher levels of recreational screen time and had greater sleep problems also experienced greater depressive symptoms. Greater disruptions to child sleep patterns was also associated with elevated anxiety and irritability symptoms, after accounting for physical activity and screen time. Similarly, poorer parent sleep quality and lower levels of physical activity were associated with poorer parent mental health, increased emotion regulation difficulties, and reduced positive affect. Given the association between the movement behaviours and indicators of mental health for both children and parents during the early stage of COVID-19; coupled with the finding of mostly unfavourable differences in these behaviours compared to pre-pandemic rates, there is potential that adversity associated with the COVID-19 pandemic may have long-term effects on the development of psycho-cardiologic diseases. While longitudinal investigations of this cohort are needed to confirm this hypothesis, at the very least, this study identifies a number of potential modifiable levers that may reduce both risk for more proximal mental health problems and more distal CVD risk.

Australian movement behaviour guidelines recommend that adults be active on most, and preferably all, days of the week, and that children should accumulate 60 min or more of moderate to vigorous physical activity per day, involving mainly aerobic activities (3). During the pandemic, children were engaging in 5 days of at least 30 min of moderate or vigorous physical activity, slightly below recommended levels. This finding is somewhat consistent with recent Canadian online survey showing that only 18% of children were adhering to physical activity recommendations (50). Results are also consistent with Moore et al. who reported in their online survey that children experienced a significant decline in all physical activities compared to pre-pandemic rates, with the most dramatic decline seen in outdoor physical activity and sport (16). Whether the lower rates of child physical activity during the early stages of COVID-19 in the current cohort compared to pre-pandemic rates is maintained beyond the initial lockdown restrictions will be important to determine. Physical activity is a known primordial risk factor for later CVD development (51), therefore, any maintenance of low levels of physical activity among our cohort would be of concern. Targeted public health interventions, sympathetic to the COVID-19 context may be required to re-engage children in daily physical activity to achieve pre-pandemic participation rates.

Although parents in the current sample appear to have high levels of physical activity compared to national data, this was still lower than recommended guidelines. Even so, this increase compared to pre-pandemic national data is somewhat heartening, given lower levels of physical activity were associated with poorer mental health across all indicators, even when accounting for sleep quality. Prioritising regular physical activity may be an important strategy for improving parents' mental health, and with COVID-19 related restrictions, it may be that parents are finding more time to engage in physical activity, which may come from greater flexibility in employment conditions or commute time. These higher rates of physical activity among parents are also heartening in terms of the more distal risk for later CVD development, given insufficient levels of physical activity is considered a primordial risk for later CVD (51). Further investigation into the contributing factors that may explain these trends among parents would be worthwhile for public health efforts aiming to improve physical activity levels among the general population, which may in turn improve proximal indicators of mental health indicators but also more distal risk for CVD. However, given the cross-sectional nature of the data, the reverse association cannot be ruled out—that instead, poor mental health contributes to lower physical activity. Regardless of the direction of associations, a key issue however, is that this increase in physical activity did not translate to increases in child physical activity behaviour. Surprisingly, increased physical activity in children was associated with higher levels of irritability. It may be that parents of children with high irritability may be prioritising exercise as an outlet to manage difficult child behaviour.

A growing body of research has demonstrated an increase in sleep problems in the general population as a result of the COVID pandemic (52–56), however, these studies have not systematically compared rates of sleep problems to pre-pandemic levels and/or have not focused on parents and children specifically. The higher levels of child sleep problems and irregular bedtime reported by parents in the CPAS cohort, and the connexion between child sleep problems and indicators of poorer mental health, suggest that sleep has the potential to be an important modifiable factor to improve children's functioning during the COVID-19 pandemic. Consistent with this notion, pre-pandemic studies have connected poorer sleep to increased mental health difficulties in children (57). Even though it may be tempting to relax the rules around children's bedtime during lockdown restrictions, our data suggest that sleep problems during the pandemic continue to be associated with worse mental health in children. Parents in the current study also had substantially higher rates of sleep problems compared to national data, which is consistent with findings from a cross-sectional study of 1,005 adults in France (52). It is notable that over half of parents rated their sleep quality as problematic. Moreover, sleep problems were associated with poorer parent mental health across multiple domains, even when accounting for physical activity levels. This is consistent with the findings of Stanton et al. (55) in their general Australian sample of adults, however, we extend these findings by considering parents specifically, and simultaneously accounting for physical activity levels.

We found mixed evidence concerning screen time. Children had lower levels of recreational screen use during weekdays but higher levels on weekends compared to pre-pandemic national data. It is important to note that parents reported on their child's screen time related to “non-educational purposes.” It may be that our findings are affected by variability in the context of schooling across participants. For example, a portion of children are likely to have been on school holidays between late March and late April (varying by State/Territory), overlapping with our data collection period (8th−28th April 2020). Further, about half the sample were juggling work from home while supervising children, and a portion of these children were engaged in remote learning (“home-schooling”) at the time of data collection; which is likely to bear an increased screen time load compared to on-campus school learning. It may be that parents restricted non-educational screen time for children already spending much of their day on screens for educational purposes.

In unadjusted analyses, increased weekday and weekend screen time was associated with child depression symptoms but this association was attenuated for weekday screen time when accounting for sleep problems, physical activity and other covariates. Similarly, associations between weekend screen time and child depression symptoms were attenuated when restricting analyses to those aged 6 years and above. Global movement behaviour guidelines for children and adolescents (4) recommend that young children aged 3–4 years engage in no more than 1 h of sedentary screen time, and that older children and adolescents 5–17 years engage in no more than 2 h of sedentary recreational screen time. On average, children in our study were engaging in 3 h of screen time on weekdays and 4 h on weekends, above recommended guidelines.

It is important to note that children may be using screens to maintain social connexions and this likely has many positive benefits, particularly in the context of limited opportunities for in-person interactions given COVID-19 restrictions. It is also difficult to tease apart the use of screens for educational and non-educational purposes. Overall, the lack of association between screen time and poor child mental health is reassuring for parents. Further, it may not be realistic to reduce child screen time during COVID-19 social distancing restrictions. Instead, family media plans can be used to manage screen time at home and parents should also consider the quality of the content that children are accessing during screen time (58). Fading (graduated extinction) approaches can gradually reduce non-educational screen time to recommended levels. Overall, it will be important to monitor screen time in this cohort to see whether screen time naturally reduces once restrictions have lifted.

Surprisingly, we found little evidence that COVID-19 related environmental or psychological stressors moderated associations between movement behaviours and mental health outcomes in children. However, for adults, COVID-19 environmental stressors moderated the association of sleep quality with both depression and emotion regulation. This is consistent with emerging research linking COVID-19 related risk factors to increased sleep difficulties (53). Prevention and intervention efforts that support parents to navigate environmental stressors related to the pandemic need to move beyond helping individuals cope. Focussing on the broader policy levers that may alleviate such stressors, including policies related to financial aid, secure housing and employment, access to COVID-19 safe childcare and school environments, and gendered division of labour will likely have a greater impact on alleviating COVID-19 related stressors and in turn improve parent sleep and mental health. Future research would benefit from focussing on these systemic, policy-based interventions.

Taken together, our findings linking disrupted sleep, low levels of physical activity and weekend screen time to a range of mental health indicators may have important implications for later psycho-cardiological disease. An emerging body of evidence in children and adolescents suggests that early experiences and environments (59, 60) in general may influence the course of health and chronic illness. COVID-19 related stressors and the experience of early depression and other emotional distress in children may be particularly relevant to the course of chronic disease development (60, 61). A compelling body of evidence has linked both stress and depression to a range of CVD risks and diseases (8, 62–68). Understanding the trajectory and prevailing nature of the COVID-19 related stressors and mental health problems reported by parents in the current study has particular importance when considering risk for later CVD. It has previously been suggested that persistent adversity and ongoing chronic stress may result in the greatest risk to CVD and that this increased “wear and tear” on the cardiovascular system over time, induced by stressful experiences that over use and dysregulate stress response pathways that is most problematic (66, 68). In addition, better quality sleep and higher levels of physical activity are also associated with a lower risk for developing CVD and better prognosis among those with established CVD (12, 69). It is through continuing longitudinal studies such as the CPAS study, and others around the world that the clinical significance of COVID-19 related stressors both in terms of proximal mental health problems and in terms of risk for more distal CVD will be better understood.

Our study has several strengths. The samples are relatively large, and included both children and parents. Our study is comprehensive in that we examine multiple domains of mental health and movement behaviours. We were further able to compare movement behaviours to nationally representative pre-pandemic data. However, several limitations should be considered. First, our sample was recruited online and appears to be more vulnerable than the general population. One example is that the rates of ADHD and ASD are much higher than would be expected in the general population. To address this, we applied sample weighting and conducted a sensitivity analysis indicating that our results are robust, both with and without the higher-risk ADHD/ASD children. Second, given that this is a cross-sectional study directionality and causality cannot be inferred and it is possible that the investigated associations are in fact, bidirectional, whereby poor mental health influences sleep, screen time and physical activity. However, population-based longitudinal research has shown that sleep difficulties appear to precede internalising difficulties in childhood, while there is more evidence of a bi-directional association between sleep and behavioural difficulties (57). Longitudinal examination of this cohort will enable further understanding of the directionality between movement behaviours and mental health. Finally, the role other important risk factors for both mental health and CVD risk, including diet and alcohol use, could not be considered as no data was collected on these constructs.

In summary, children in the current study had higher rates of sleep problems and increased weekend screen time compared to national pre-pandemic data. Parents had higher levels of sleep problems compared with pre-pandemic data, while physical activity also appeared to be higher. Poor sleep in both parents and children, and lower levels of physical activity and higher levels of weekend recreational screen time were associated with poorer mental health outcomes. Monitoring this cohort over time will be important to examine whether changes in movement behaviour are enduring or naturally improve with the easing of restrictions and whether these changes have enduring effects on either parent or child mental health, and in turn, more distal risk for psycho-cardiological disease.

## Code Availability

CPAS code is shared on the Open Science Framework.

## Data Availability Statement

Data from CPAS participants who consented to data sharing (91.21%) will be available through the Australian Data Archive (currently under review) for research projects that are related to CPAS. Please direct enquires to the corresponding author, lisa.olive@deakin.edu.au.

## Ethics Statement

The studies involving human participants were reviewed and approved by Deakin University Human Ethics Advisory Group (Project No: HEAG-H 52_2020). The patients/participants provided their written informed consent to participate in this study.

## Author Contributions

LO conceived the current study, while EW conceived the larger CPAS study. LO, EW, and ES drafted the initial manuscript. EH and TB were responsible for data management. LO, EW, TB, EH, and RT completed the statistical analysis. All authors contributed to and provided feedback on the final draft.

## Funding

This work was supported by Deakin University, Centre for Social and Early Emotional Development, School of Psychology, Faculty of Health, Victoria, Australia. LO (1158487) and EH (1156909) are supported by National Health and Medical Research Council (NHMRC) Early Career Fellowships. DH was supported by a NHMRC Investigator Grant (1197488). ES was supported by an NHMRC Career Development Fellowship (1110688) and a Veski Inspiring Women's Fellowship. MB was supported by a NHMRC Senior Principal Research Fellowship (1156072).

## Conflict of Interest

The authors declare that the research was conducted in the absence of any commercial or financial relationships that could be construed as a potential conflict of interest.

## Publisher's Note

All claims expressed in this article are solely those of the authors and do not necessarily represent those of their affiliated organizations, or those of the publisher, the editors and the reviewers. Any product that may be evaluated in this article, or claim that may be made by its manufacturer, is not guaranteed or endorsed by the publisher.
